# Modern Medicine: Towards Prevention, Cure, Well-being and Longevity

**DOI:** 10.4103/0973-1229.58817

**Published:** 2010

**Authors:** Ajai R. Singh

**Affiliations:** * *Editor, MSM*

**Keywords:** *Medicine*, *Eros*, *Thanatos*, *Disease*, *Prevention*, *Cure*, *Palliation*, *Longevity*, *Well-being*, *Centenarians*, *Meditation*, *Yoga*, *Complementary and Alternative Medicine*, *Pharmacology*, *Psychopharmacology*

## Abstract

Modern medicine has done much in the fields of infectious diseases and emergencies to aid cure. In most other fields, it is mostly control that it aims for, which is another name for palliation. Pharmacology, psychopharmacology included, is mostly directed towards such control and palliation too. The thrust, both of clinicians and research, must now turn decisively towards prevention and cure. Also, longevity with well-being is modern medicine’s other big challenge. Advances in vaccines for hypertension, diabetes, cancers etc, deserve attention; as also, the role of meditation, yoga, spirituality etc in preventing disease at various levels. Studies on longevity, life style changes and healthy centenarians deserve special scrutiny to find what aids longevity with wellbeing. A close look at complementary and alternative medicine is needed to find any suitable models they may have, cutting aside their big talk and/or hostility towards mainstream medical care. Medicine is a manifestation of the human eros, and should not become a means of its thanatos. It must realise its true potential, so that eros prevails, and thanatos prevails only ultimately, not prematurely.

## Introduction

I got up with a dream about medicine and was thinking vaguely of analyzing it. And then I thought why, what the heck. Freud is no longer around, and psychoanalysis is not current coin. And life is moving at its nice, leisurely pace, anyway. No major hassles. So why dig?

But a previous early morning dream-realization continues to linger. Let me tell you a bit about it. Modern medicine has progressed, and we must thank the whole bunch of researchers and clinicians for it. But so has pathology. It is not that the number of diseased has reduced, nor total quantum of distress related to it. Diseases, and the afflicted, are not reducing in numbers; they are only changing in type (Singh and Singh, 2005). No doubt, we have increased longevity, made old age livable, reduced infant mortality, made everyday living itself more distress-free by the numerous medicines/procedures in our armoury; but we have not reduced the number of diseased, nor found cures for any diseases except the infectious; as also of course some remarkable successes with emergency care. [Both, great mobilization of resources and energies towards reducing social distress. Just as an animal, human included, will struggle and strain to cope with emergencies, especially a threat to life or limb, similarly as though the society-animal has developed efficient methods to cope with its emergencies. So methods to tackle infections and acute medical/surgical problems are really efficient.]

In the case of most other medical care, it is mainly control -- slowing advance, reducing distress, minimizing disability, prolonging death.

It is passé to think in terms of cure, as though it were a hate-word.

## Modern Medicine Mostly Palliative

Modern medicine is mostly palliative, and rather proud of it. By palliation, I am not talking of cancers. I mean reducing or easing the severity of a pain or a disease without removing the cause, or effecting a cure (from Latin palliare, to cloak).

In other words, control.

It is rather like making a virtue out of a necessity and also, false bravado.

What medicine needs to do is propel towards cure on one hand and prevention on the other. Cure, however, is out of consideration, because it appears unachievable, and prevention is nowhere in the collective consciousness of biomedical researchers and opinion makers, I suspect probably because it would ultimately make them redundant. And the cure and prevention lobby makes only weak noises, if at all, and does not have the clout to change mainstream opinion.

What this ultimately means is that while the truth is there for all to see, and work over, it does not get done. Why should it be so? Is it because mankind is propelled by a recklessly driven *thanatos*, as Freud said? That it will deny, and prevent realizations that matter, because it must propel itself towards decay and ultimate death, even as it ostensibly appears to be fighting it all the time by its many heroic and sustained manoeuvers?

Let me put it a little differently. What should the modern doctor be doing? He should either prevent a disease so it does not occur, or cures it if it does. What does the modern doctor, me included, do? He neither prevents, nor cures in any but a few conditions. He only controls spread of the disease, and palliates while so doing. His motto can be captured in a sentence: To cure sometimes, to comfort always, to hurt the least, to harm never (Singh and Singh, 2005).

The epitome of palliation, true, but not of cure, or prevention.

This realization does not propel him towards cure or prevention. It propels him towards greater and more efficient palliation. As though to quickly rid him of the realization that must stare him in the face, and expose his nakedness before all, and most importantly, his own consciousness.

## What Do We Do? *Eros* Guides, *Thanatos* Ultimately Prevails

So what do we do? It would be convenient, but facile, to accept Freud’s formulation. That *thanatos* and *eros* are the two instincts that rule our life, and that *thanatos* does, and must, ultimately prevail, for the ultimate reality is death, whatever manoeuvers one may carry out to prevent it. Which explains why modern medicine’s propulsion is towards control, not cure or prevention: it is just one manifestation of this *thanatos*.

That of course, does not mean we do not exercise *eros*, for it will exercise itself anyway. And medicine’s palliative goals are but one manifestation of this exercise.

This is an effective, but weak, *eros*. But in not making cure and prevention its goals, medicine as though accepts the inevitability of a *thanatos* to rule over its efforts.

Medicine, on the other hand, has to become the most glorious manifestation of an *eros* that prevents/cures disease and promotes well-being in its best possible form. This will be an effective, and strong, *eros*.

Till death occurs. Which it will.

When, ultimately, *thanatos* prevails.

Which can lead us to ask the question: If ultimately death had to prevail, why exercise *eros* at all? Why not accept the reality and stop fighting *thanatos*, rather accept it willingly?

The answer is that *thanatos* prevails only *ultimately*. It is the interim which really matters - the interval between life and death. What we do with it alone is in our hands, and that’s where the whole human effort is directed, and legitimately so. Hence *eros* must be consciously, and vigorously, promoted. Why allow what must occur only at the end to override this process?

The key concept is *ultimately*. Not prematurely, not unobtrusively as it does today, due to our lack of directedness.

As also the realization that if ultimately *thanatos* must prevail, one must know when to give way, and stop struggling too, to ensure a dignified exit. The whole expenditure of human resources to avoid the inevitable when it has to occur will then stop. Modern medicine’s role of helplessness mixed with grandiosity that promotes life at any cost e.g. in persistent vegetative states, will then end.

An *eros* must guide our consciousness, and our efforts, even as we accept the truth of a *thanatos*, which must *ultimately* prevail.

## Prevention and Cure, the Watchwords

If we accept this, the path before us is very clear.

### Prevention

The first goal of medicine is to see to it that no one has to reach a hospital or a clinic. This is what I mean by prevention. [Technically called primary prevention; not of course ruling out the importance of secondary prevention (early detection and prompt treatment) and tertiary prevention (restoring function and reducing disability)]. This should be the very first goal, not the last; which means, health promotion/education activities along with clean water, nutritious food, clean and disaster free habitation, proper sanitation, control of pollution, poverty alleviation, empowerment of the deprived and disadvantaged, life-style modifications. A tall order, which involves multiple agencies, not in control of medicine and its movers. That is one prime reason why it is not top of the agenda, perhaps. And of course because it cuts at the very root of the justification for the medical establishment, and its proliferation. But medicine has as much a treatment orientation as a social perspective. For health is a means to well-being, and health can be achieved for all only when all are mobilized for health and become conscious of what should be its legitimate thrust (Singh and Singh, 2004). Moreover, prevention, understood as preventing a disease from occurring, also means, finding out vaccines, and other preventive measures, for all its diseases, not just the infectious. Let this stop sounding ludicrous. It is comforting to note that work on vaccines is underway, especially for diabetes (Phillips *et al*., 2008; Richardson *et al*., 2009), hypertension (Mitka, 2007; Ambühl *et al*., 2007; Phisitkul, 2009), cancers (especially cervical: Jenkins, 2008; Keam and Harper, 2008; Schwarz, 2008), and at least suggested for schizophrenia and other mental disorders (Tomlinson *et al*., 2009). And, related to this, it must research and highlight life style changes which prevent disease, and tackle diseases of poverty and of lifestyle (Singh and Singh, 2008). Some work in this field is already on, for example, in cancers (Anand *et al*., 2008), Type 2 Diabetes (Misra, 2009), cardiovascular disease (Pischke *et al*., 2008), ulcerative colitis (Langhorst *et al*., 2007); as also the beneficial effects of a vegetarian diet in lifestyle diseases (Segasothy and Phillips, 1999). Preventive psychiatry also needs a boost, for psychiatry, and overall for medicine, for do we not know that psychological distress is at the root of common medical problems that reach a primary care physician, and complicate many manifestations of other disorders at all levels of their manifestation. The complex relationships between gene-environment interactions, particularly the interplay of vulnerability and resilience factors within a person’s biography need close scrutiny in individualized preventive psychiatry (Müller-Spahn, 2008), as does reduction of stigma in secondary prevention (Reeder and Pryor, 2008). The role of health psychology and the related field of behavioural medicine which focus on the interplay among biological dispositions, behaviour, and social context also need enthusiastic backing as a means to health promoting behaviours and preventing health damaging ones (Kaplan, 2009). Modern medicine must look closely at, and not pooh pooh, the claims of alternative and complementary medicine, including yoga, meditation and spirituality, just because one is put off by their tall sounding claims, and some charlatans in the group. Rather, it must submit their claims to rigorous scientific and experimental scrutiny. Some recent studies in yoga in general (Lipton, 2008; Bijlani, 2008; Corliss, 2001; Oken *et al*., 2006; Brown and Gerbarg, 2005; Shapiro *et al*., 2007; Flegal *et al*. 2007), and yoga in cancers (Culos-Reed *et al*., 2006; Bower *et al*., 2005; Smith and Pukall, 2009; Danhauer *et al*., 2009) are promising in this direction. Studies of meditation as an adjunct to modern medicine deserve special mention here. Some promising leads are in works on Longevity and health through yogic meditation (Bushell, 2009), and meditation in general (Bushell and Thiese, 2009); meditative practices for health (Ospina *et al*., 2007), and their clinical trials (Ospina *et al*., 2008); Sudarshan kriya in stress, anxiety and depression (Brown and Gerbarg, 2009); Transcendental Meditation and longevity (Alexander *et al*., 1989); meditation and slowing of aging, (Epel *et al*., 2009); mindfulness and distress (Jain *et al*., 2007); and mindfulness and well-being (Shapiro *et al*., 2008). Spirituality and its various scientific studies need a close scrutiny too. Some areas of spirituality which have interested researchers in recent times are positive emotions and spirituality (Vaillant, 2008), its neurobiology (Mohandas, 2008), healing presence (McDonough-Means *et al*., 2004), spiritual encounter and complementary treatment (Foster, 2006), spirituality and psychiatry (Mohr, 2006), health and spirituality in critical care (Puchalski, 2004), spirituality and critical care holistic nursing (Carpenter *et al*., 2008), difficulty in talking about spirituality in a medical setting (Molzahn and Sheilds, 2008) etc. To promote rigorous scientific scrutiny of claims in Complementary and Alternative medicine [CAM], laudable efforts are on by relatively new Journals in the field like Evidenced Based Complementary and Alternative Medicine (an Oxford Journal, since June 2004, http://ecam.oxfordjournals.org), BMC Complementary and Alternative Medicine (Published by BioMed Central, since 2001, http://www.biomedcentral.com/bmccomplementalternmed), Alternative Therapies in Health and Medicine (since 1995, first journal in the field of CAM to be indexed with NLM http://www.alternative-therapies.com), Journal of Alternative and Complementary Medicine (since 1995, Official Journal of the International society for Complementary Medicine Research, http://www.liebertpub.com/products/product.aspx?pid=26). Some notable relatively recent work in CAM in the field in anxiety and depression (van der Watt *et al*., 2008), depression in women (Manber *et al*., 2002), menopausal women (Kronenberg and Fugh-Berman, 2002), sleep disorders in the elderly (Gooneratne, 2008), osteoarthritis (Ernst, 2006), asthma (Pretorius, 2009) etc, should not go unnoticed. And while all this happens, the preventive and social medicine guys from mainstream medicine need to awaken and clean up their act. To stop being sidelined, and point out how they matter. And of course, to re-emphasize that prevention is better than cure (Phakathi, 2009, where it is in relation to child sexual abuse, but applicable elsewhere too).

**Figure 1:**
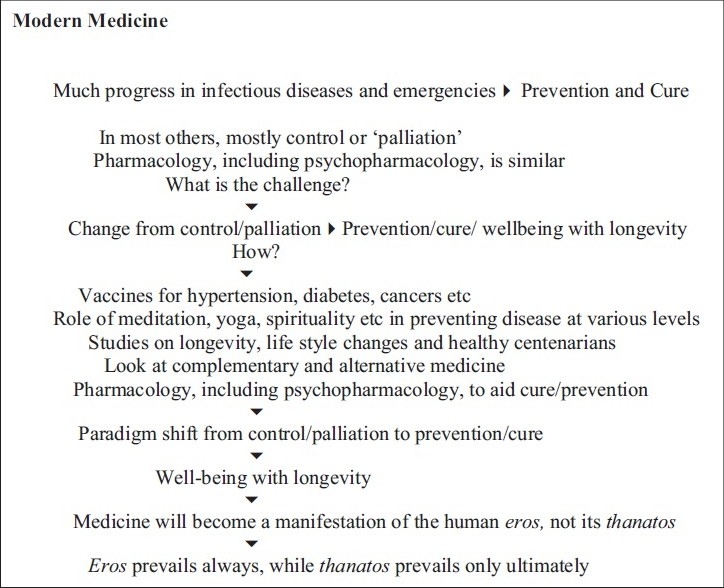
Flow chart of Paper

### Cure

The second goal is to cure disease/s if he lands up in a hospital/clinic, so the patient does not land with the same disease/s again. Or with related ones. And lands up lesser in hospitals and more at workplaces and homes. For this, the emphasis must shift from control to cure, even if we know it is a distant goal today. Distant does not make it unachievable. In fact distant must make clear how much more need be done, and in which direction. I would have loved to add a list of references here, but am sorry to find very little of any consequence.

### Longevity and well-being

The third is to closely study and report on longevity and well-being studies. Well-being implies the presence of (1) positive emotions and the absence of negative ones; (2) mature character traits, including self-directedness, cooperativeness, and self-transcendence; (3) life satisfaction or quality of life; and (4) character strengths and virtues, such as hope, compassion, and courage, all of which are now measurable by scales (Cloninger, 2008). Some relatively recent literature focuses on telomeres and longevity (Haussmann and Mauck, 2008); sex differences in longevity, (Franceschi *et al*., 2000); Immunology and longevity (Candore *et al*., 2006); psychosocial factors and longevity (Darviri *et al*., 2009) etc. The paradigm of Reorganizational healing [ROH] is an interesting recent work in the field of wellness, behaviour change, holistic practice and healing (Epstein *et al*., 2009). Anyone who has not visited a hospital and is over 60 years is a precious commodity to research. Anyone who has none of the lifestyle diseases till 60 is also similarly precious. All those who are 90 and active physically and mentally, even if diseased, form another very precious group. And all centenarians are the most precious group to study. It is fascinating to see the breadth of studies on this topic. There is a burgeoning research on centenarians in the last decade, some of promise and interest are in the areas of Centenarians and healthy aging (Engberg *et al*., 2009); antioxidants and healthy centenarians (Mecocci *et al*., 2000); nonagenarians and centenarians in China, (Ye *et al*., 2009); centenarians in Bama (Xiao *et al*., 1996); quality of life and longevity, (Jeune, 2002); Danish centenarians, not necessarily healthy but still autonomous (Andersen-Ranberg *et al*., 2001); and not necessarily having dementia, (Andersen-Ranberg *et al*., 2006); centenarians and their cognitive functions, (Silver *et al*., 2001); dementia free centenarians (Perls, 2004a, 2004b); centenarians being different (Perls 2006); cognitive states of Centenarians, (Luczywek *et al*., 2007); successful aging in centenarians: myths and reality, (Motta *et al*., 2005); physical activity and centenarians (Antonini *et al*., 2008); centenarians aging gracefully (Willcox *et al*., 2007) etc.

### Pharmacology as a tool to prevention, cure and well-being

Pharmacology is a very potent tool, but most of it is directed towards palliation and control at present. It must be marshaled towards cure and prevention. This is its big challenge, if it can transcend economic compulsions and awaken to its true role in medicine. Psychopharmacology is similar, but, additionally, it needs greater help from clinical and investigative diagnostics to help psychiatry become a full-fledged medical discipline from its interim status at present (Singh and Singh, 2009). Although it has mainly developed newer drugs that are more tolerable rather than better in efficacy, it has also helped reduce stigma and made primary care physicians more comfortable in treating mental disorders (Schwartz, 2010).

### Paradigm shift from control/palliation to prevention/cure

The thrust and focus must shift from control/palliation to cure and prevention. The ultimate goal is longevity with well-being, of which freedom from disease is a very important ingredient. Major research funding must guide these efforts, even as we do not neglect greater refinements in modern ‘palliative’ medicine.

### The master winces as he smiles

The course is clear. The goal is set.

Shall we try and get the bandwagon moving?

The other course is of course to awaken from a slumber with a dream and remember the great master and his strivings to understand the fundamentals of disease, as he talked of dream analysis and the unconscious; to quieten the realization with a denial that is so characteristic of much of modern medicine, as also pharmacology, psychopharmacology included. And lapse into a slumber that pushes uncomfortable dreams into realms that work to energize the *thanatos* and propel man to his inevitable end.

You decide what to do.

The master smiles wryly, even as he turns wincing with pain in his grave.

## Concluding Remarks

1. Let’s turn the course from palliation and control to prevention and cure. Let’s first think it’s possible.
2. This is the future goal of pharmacology, including psychopharmacology, as it is of medical practice and research.
3. To ensure longevity with well-being, let’s not discount the work of complementary and alternative medicine. Rather submit them to rigorous scientific scrutiny.
4. We also need to promote longevity studies.

### Take home message

A paradigm shift in mainstream medicine from control and palliation to prevention and cure must prevail. The ultimate goal is longevity with well-being.

### Conflict of interest

None declared

### Declaration

This is my original unpublished piece, not under consideration for publication elsewhere.

## Questions That This Paper Raises

1. What needs to be done to bring about the paradigm shift from control and palliation to prevention and cure?
2. Is cure at all possible?
3. Will mainstream medicine ever be able to incorporate the beneficial findings of CAM?
4. Will prevention and cure strike at the very legitimacy of the proliferation of the medical establishment, and therefore never assume top priority, even as we give lip service to it?
5. What needs to be done to promote wellness with longevity?
6. How do we propel medicine to become a means of an effective and *strong* eros, rather than an effective but *weak* one as at present?

## About the Author


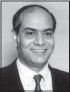
*Ajai R. Singh M.D. is a Psychiatrist and Editor, Mens Sana Monographs, (http://www.msmonographs.org). He has written extensively on issues related to psychiatry, philosophy, bioethical issues, medicine, and the pharmaceutical industry.*
